# Considering health and health disparities during state policy formulation: examining Washington state Health Impact Reviews

**DOI:** 10.1186/s12889-019-7165-7

**Published:** 2019-07-03

**Authors:** Keshia M. Pollack Porter, Ruth Lindberg, Arielle McInnis-Simoncelli

**Affiliations:** 1 Department of Health Policy and Management, Bloomberg School of Public Health, Johns Hopkins University, 624 North Broadway, Rom 380A, Baltimore, MD 21205 USA; 2 Health Impact Project, The Pew Charitable Trusts, 901 E Street NW, Washington, DC 20004 USA; 3 2018-2019 U.S. Fulbright Student Program (Pre-Doctoral Research), Barcelona, Spain

**Keywords:** State policy, Health in all Policies, Legislation, Disparities, Public health

## Abstract

**Background:**

As part of efforts to expand Health in All Policies (HiAP) in Washington State in the U.S., the Washington State Board of Health (BOH) received statutory authority in 2006 to conduct Health Impact Reviews (HIRs). HIRs analyze the potential impacts of proposed legislation and budget decisions on health and health disparities. Public health professionals who are aware of HIRs are interested in adopting a similar process in their states; however, there is limited information about HIRs, how they are perceived, and how they could advance HiAP.

**Methods:**

This research involved a descriptive analysis of a sample of HIRs and semi-structured interviews with a purposive sample of 17 key informants. For the descriptive analysis, all HIRs requested or completed between January 1, 2007 and April 1, 2016 that had a request form submitted by a legislator or the governor that was available in the BOH’s online database were reviewed. Information was collected on several variables including the bill number and title, sponsor and political affiliation, and the sector to which the bill or budgetary proposal pertained. A purposeful sample of legislators, staff, advocates, and lobbyists who were involved with HIRs during the study period were invited to participate in semi-structured interviews. Topic coding was used to identify key themes from the qualitative data.

**Results:**

During the study period, 20 legislators requested 36 HIRs; 32 HIRs were completed. HIRs were requested for several bill topics, including education (11/36) and labor and employment (9/36). Legislators who requested HIRs felt they provided valuable data on health and health disparities for proposed bills. Individuals who were less supportive of HIRs perceived them as an advocacy or political tool. The main barrier to widespread use of HIRs in Washington was a lack of awareness among legislators.

**Conclusions:**

HIRs are one strategy to advance HiAP for state policy decisions. HIRs are a potentially effective tool for highlighting how legislative proposals and budgets positively and negatively impact health and health disparities. Future efforts should promote awareness and highlight shared benefits of HIRs among legislators and their staff, as well as their scientific integrity, methodological rigor, and objectivity.

**Electronic supplementary material:**

The online version of this article (10.1186/s12889-019-7165-7) contains supplementary material, which is available to authorized users.

## Background

It is widely recognized that many of the determinants of health, equity, and well-being originate from decisions beyond those in the health sector [1]. Thus, policies formulated and implemented by sectors such as transportation, housing, and criminal justice can have significant impacts on determinants of health and well-being, and health outcomes. Health in All Policies (HiAP) is one strategy, gaining popularity in the U.S., to consider how policies from a range of sectors can integrate health considerations into decision-making. HiAP is an “approach to public policies across sectors that systematically takes into account the health implications of decisions, seeks synergies [across sectors], and avoids harmful health impacts in order to improve population health and health equity” [2]. HiAP initiatives, although varying in their design and implementation, inherently focus on the routine consideration of health in decision-making and changes to organizational structures to promote health and health equity.

Various strategies to achieve HiAP include promoting the use of health impact assessments (HIAs); conducting health and equity lens analyses; creating a task force representing multiple sectors to review and propose policies and programs; or integrating health considerations into planning documents [2, 3]. HiAP approaches have been promoted and reinforced globally through, for example, the 2011 Rio Political Declaration on Social Determinants of Health, 2014 Helsinki Statement on Health in All Policies, and most recently, as part of the United Nations Sustainable Development Goals [4–6]. Examples of HiAP approaches include Thailand’s National Health Act 2007, which established the National Health Commission as an advisory body to the Cabinet on health policies and strategies and the Government of Quebec’s whole-of-government approach to health, which involves 15 ministries and government agencies from different sectors working together to achieve population health goals [6].

Within the U.S., several states have explored the opportunity to advance HiAP through legislation that requires or facilitates a HiAP approach. An analysis of state bills and laws identified 28 HiAP bills that were introduced in state legislatures between January 2012 and December 2016, and found that eight states and the District of Columbia had enacted or amended a HiAP law as of December 31, 2016 [7, 8]. Another important opportunity to advance HiAP in state governments is through the examination of proposed legislation across a range of sectors. In 2006, the Washington state legislature authorized the Washington State Board of Health (BOH), in collaboration with the Governor’s Interagency Council on Health Disparities, to conduct Health Impact Reviews (HIRs) in support of HiAP in the state’s legislative process. HIRs use “existing published literature, data, and/or expert opinion to provide an objective, evidence-based analysis of a proposed legislative or budgetary change to determine its likely impacts on health and health disparities” [3]. HIRs consider the potential impacts on health outcomes and health disparities by examining differences in disease, death, and other adverse health conditions that exist between populations, such as differences between low- and high- income populations. HIRs are conducted upon the request of a member of the legislature or the governor. HIRs typically are subject to time constraints; BOH must complete the HIRs within 10 days when requested during the legislative session.

HIRs involve creating a logic model depicting possible pathways leading from the provisions of the bill to health outcomes and conducting a literature review of each pathway [9]. The BOH also assesses the strength of evidence for each pathway based on established criteria. HIRs include an executive summary, a description of key health impacts, a summary of findings, the strength of the evidence in the research literature, perspectives from key stakeholders, and an annotated bibliography. The BOH also consults with experts and stakeholders with diverse perspectives on the bill to learn about important contextual information.

From the Washington state HIR process’ inception at the beginning of the 2007 legislative session through the end of the 2017 session, the BOH completed nearly 60 HIRs [9]. Because of the recession, funding for HIRs was suspended in 2009, which meant there was no support for staff to conduct them until the economy improved and funding was restored in 2013.

Across the U.S., states are expanding HiAP efforts [3, 7, 8]. Although HIRs are intended to be one way to advance HiAP, there is limited information about HIRs, how they are perceived, and how they could advance HiAP. To address this gap in knowledge, this article documents findings from the first independent exploration of HIRs by: (1) conducting a descriptive analysis of HIRs; and (2) exploring how legislators and other key stakeholders perceive them, including their impacts and consideration of health disparities and inequities.

## Methods

This research involved two components: a descriptive analysis of a sample of HIRs and qualitative data collection to understand perceptions of HIRs. For the descriptive analysis, the team reviewed all HIRs requested or completed between January 1, 2007 and April 1, 2016 that were listed in BOH’s online database [9], and at a minimum, had a request form submitted by a legislator or the governor that was available in the database. For these HIRs, the team reviewed the completed request form and full HIR report, if available, to identify the: bill number and title or title of budget proposal; companion bill numbers; the legislator who requested the review and his or her political affiliation; the date BOH received the HIR request; the due date for the full HIR report; the legislative session; the topic of the legislative or budgetary proposal; the sector to which the bill or budgetary proposal pertained; bill sponsors and their political affiliations; key findings from the HIR; the number of references cited in the review; the outcome of the bill or proposal; and the number of stakeholders included in the request form. One member of the study team abstracted the data, and a different member of the team randomly selected and reviewed 10% of the HIRs (4/36) to validate the sample. Data were entered in Microsoft Excel.

The qualitative data collection included a nonrandom sample of 17 individuals that were recruited in two phases. The team first used snowball sampling to identify individuals who conducted HIRs [10], as well as partners—such as staff from other government agencies or lobbyists—who were engaged as stakeholders while an HIR was conducted, used data from an HIR in testimony, or helped develop or advocate for a bill that was the subject of an HIR. This sampling strategy yielded seven interviews with nine interviewees (four state government employees, three lobbyists, one former legislator, and one advocate). Two members of the team conducted these interviews in-person in Washington state, using a semi-structured instrument that asked about the interviewees’ knowledge of, experiences with, and perceptions of HIRs; and when relevant, questions about the origins of the HIR process were included (instrument included as an online supplement) (Additional file 1). These seven in-person interviews lasted between 30 and 60 min.

The team initially sought to survey all 147 Washington state legislators via an online survey. All legislators and their legislative assistants received the online survey, but the response rate was low (less than 10%), and those who responded lacked familiarity with HIRs. Thus, to capture legislator perspectives, the team conducted a second phase of sampling for the qualitative interviews by emailing requests for interviews to 16 legislators and staffers from both chambers and political parties, as well as lobbyists who represent the interests of both political parties. These individuals were identified through nonrandom snowball sampling using suggested contacts provided by the initial nine interviewees.

Of the 16 people who were invited to participate in the second phase of interviews, eight agreed (six legislators, one lobbyist, and one committee staff member). Of the eight people who did not participate in interviews, the team received no response from five of them; one legislative aide offered to speak with the team on behalf of the legislator but did not have enough knowledge about HIRs; and two legislators were unavailable for an interview during the study period. These interviews occurred using a slightly modified version of the previously mentioned instrument. For those who had no experience with HIRs, the interviewers shared basic information about HIRs and asked interviewees about their perceived utility, challenges, effectiveness, and how to increase awareness of HIRs. These interviews also lasted between 30 and 60 min.

The second set of interviews were conducted by two members of the study team via phone who independently captured notes that were later combined for each interviewee. Interviews conducted via telephone were not recorded due to preference from legislators not to record the conversations. The Johns Hopkins Bloomberg School of Public Health approved this study and oral consent was obtained from all participants.

### Data analysis

Data from the document review were descriptively summarized in accordance with the aims of the descriptive aspect of this study. Interviews that were audio recorded were transcribed verbatim by a professional transcription company. To analyze the interview data, the study team read the transcripts and notes from conversations with the 17 interviewees. A deductive approach, informed by the study’s research questions, aims, and objectives, was used to initially develop the codes. The study’s lead investigator also read each of the transcripts and notes, and added a few new codes that emerged from this process to develop the final codebook. One randomly selected transcript was used to test the codebook; this process resulted in no changes to the codebook.

The transcripts were uploaded to Atlas.ti, a qualitative data management and analysis software. While multiple members of the team had input on the codes, the study’s lead author led the analysis of the data, which involved descriptive coding to “attribute a class of phenomena to a segment of text” [11]. Because a semi-structured interview guide was used for these interviews, consistent with qualitative data analysis procedures, the number of responses for each of the themes is not presented, rather the data are qualitatively summarized.

Several strategies were used to enhance credibility of the qualitative data: meticulous record keeping; including rich and thick verbatim descriptions of the interviewees’ experiences to support the key findings; engaging with the study team during data interpretation to reduce research bias; and when possible comparing the findings from the interviews with completed HIRs in support of data triangulation [10]. The study team did not analyze the online survey data due to insufficient data.

## Results

**Fig. 1:**
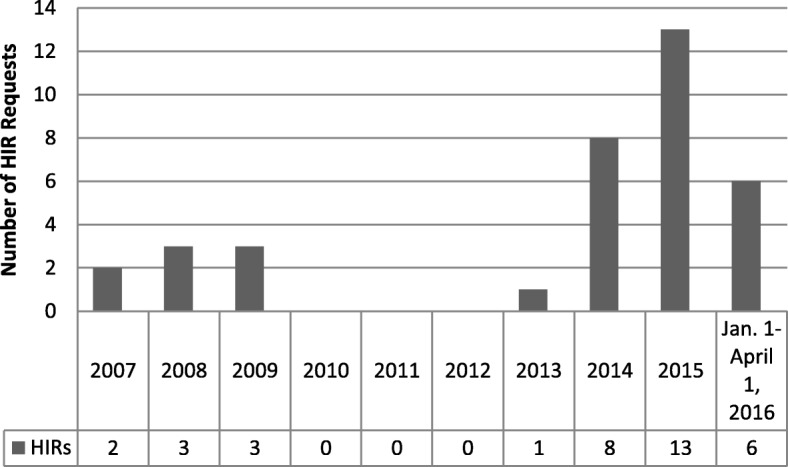
Number of HIR requests by year, 2007–2016. Source: Analysis of data from a database based on information from http://sboh.wa.gov/OurWork/HealthImpactReviews for the defined study period. Notes: Funding support for HIRs was suspended in 2009, and there were no dedicated staff to conduct them until October 2013, when funding was restored. Therefore, 2009 and 2013 represent years with limited staffing and capacity to support HIRs. In Washington, odd numbered years are long sessions (105 days) and even numbered years are short sessions (60 days), which could affect the number of requests per year

As shown in Table 1, the final sample for this descriptive analysis of HIRs included all 36 HIRs requested through April 1, 2016, of which 32 (89%) were complete at the time of the review. Two of the reviews (6%) were not yet complete, and two (6%) of the requests were withdrawn before the BOH could complete the review. Thirty of the requests (83%) focused on proposed legislation, five (14%) focused on budget proposals, and one (3%) focused on implementation decisions of a state agency. Figure 1 displays the number of HIR requests by year, accounting for the gap from May 2009–October 2013 when HIRs were suspended because of funding limitations.

**Table 1:** Summary of Health Impact Reviews reviewed in the study sample

Bill, HIR year, and sector	Bill summary	HIR findings
(Budget Proposal, 2007) Education	Would provide grants to school districts or partners increase dropout services for at-risk students.	The program has the potential to decrease health disparities if it is designed to reduce health disparities among minority students.
(Budget Proposal, 2007) Education	Would provide funding for the Office of Superintendent of Public Instruction for Financial Incentives to Attract Excellent Teachers for Hard-to-Staff Schools and Subjects to improve students’ test scores by hiring experienced teachers in low-performing schools.	The program could reduce health disparities among a large population of minority students if the salary incentives were focused in those communities.
(S.H.B. 1675, 2008) Disaster preparedness and recovery	Would require state agencies to provide bilingual or multilingual notices of public health, safety, or welfare risk when 5% or more of the residents in an affected area speak a language other than English and have limited English proficiency.	The bill had the potential to reduce health disparities faced by limited English proficiency populations, likely to be Hispanics and Asian and Pacific Islanders, resulting from emergencies and disasters.
(S.H.B. 2884, 2007, 2008) Education	Would limit the use of chemical, mechanical, and physical restraint against students in public schools. The bill would also have added a requirement research-based, school-wide, positive behavior intervention supports are included in classroom management trainings.	The health impact would be limited given the infrequently used discipline practices this bill addresses.
(H.B. 3221, 2007–2008) Economic policy	Would establish a ten-member financial services intermediary, which would work with financial institutions and community-based asset building coalitions to improve access to mainstream financial products, establish individual development accounts, and offer financial education for low-income individuals.	The bill could reduce health disparities for low-income communities.
(Budget Proposal, 2009) Health care and social services	Would eliminate the General Assistance-Unemployable program and reduce funding for subsidized health care for children in poverty, and the universal vaccine program and HPV vaccine.	The budget cuts would disproportionately impact low-income families, racial and ethnic minority communities, and women, which would likely lead to an increase in health disparities experienced by these groups.
(Implementation of Core 24 High School Graduation Requirements, 2009) Education	Would increase graduation requirements from 19 to 24 credits for high school students, including three years of math, more English, and a career concentration.	The request for an HIR was withdrawn.
(H.B. 1341, 2009) Education	Would remove state assessment as a pre-requisite for high school graduation, and would dedicate subsequent savings to assessing incentive programs for students to meet state standards and pursue higher education.	More research is needed to understand the connection between school exit exams and health determinants such as dropout and graduation rates.
(S.H.B. 1680, 2013) Education	Would incorporate opportunities to close the educational achievement gap by addressing disciplinary strategies, educator cultural competency, subgroup academic achievement, and occupational pathways.	The bill has the potential to positively impact student achievement and health among minority students by addressing educational and social needs.
(S.S.B. 6439, 2014) Education	Would update and enforce training requirements for anti-harassment, intimidation, cyberbullying, and bullying policies for implementation by district Compliance Officers.	The bill has the potential to reduce bullying and adverse health outcomes experience by those disproportionately impacted by bullying, including LGBTQ youth and students experiencing both under and overweight issues.
(H.B. 2451, 2014) Health care and social services	Would expand the list of acts that constitutes unprofessional conduct by a licensed health care provider to include performing sexual orientation change efforts on a patient under the age of 18.	The bill has the potential to mitigate harms and improve health outcomes among LGBTQ patients.
(Budget Proposal, 2014) Health care and social services	Requests 25% of the funding needed to build five community health centers to provide care to 42,300 patients.	Health disparities may be reduced among this population by increasing their access to culturally and linguistically appropriate health care.
(S.B. 5571, 2014) Health care and social services	Would require the Department of Social and Health Services to increase awareness of mental health illness and its consequences through a public awareness and education campaign targeted at those who disproportionately experience negative mental health outcomes, stigma, and barriers to care.	The bill has the potential to decrease health disparities among the target group by increasing knowledge of mental health issues, decreasing stigma, and educating the community about positive behavior changes, such as seeking help.
(S.S.B. 6554, 2014) Health care and social services	Would require personal emergency response system companies to provide the location and known medical conditions of their costumers when requested by first responders during an emergency.	The bill could improve health outcomes during an emergency for older adults, individuals with disabilities and chronic conditions, and individuals with limited mobility.
(Amendment #910 to S.B. 6552, 2014) Education	Would improve student success by increasing instructional hour and graduation requirements.	As shown on the BOH HIR website, a full HIR was not completed following the request form, “Board of Health staff are currently working with the requestor to establish a completion date for this review. This is expected to be a long-term project.”
(H.B. 2321, 2014) Labor and employment	Would create two new mid-level dental professions to practice under supervisions of dentists and in specified care settings.	The bill could improve oral health and health outcomes for low-income communities of color and those with chronic conditions.
(S.B. 6170, 2014) Labor and employment	Would require disciplining authorities specified by the state legislature to adopt rules requiring health professionals to receive cultural competency continuing education identified by the Department of Health.	The bill had the potential to decrease health disparities by improving health and health care outcomes for diverse patient populations.
(H.B. 1080, 2015) Education	Would restore funding to the health professional loan repayment and scholarship program fund.	The request for an HIR was withdrawn after the Health Professional Loan Repayment and Scholarship Program Fund was refunded in the 2015–2017 operating budget.
(H.B. 1295, 2015) Education	Would require high-needs schools without 70% of free or reduced-priced meals to offer breakfast after the bell that meets federal standards. Training for implementation of this program would be provided by the Office of the Superintendent of Public Instruction.	The bill could narrow educational and income gaps, and decrease health disparities in these schools.
(H.B. 1671, 2015) Education	Would authorize health care practitioners to administer, prescribe, and dispense opioid overdose medication to any person who may be present at an overdose - law enforcement, emergency medical technicians, family members, or service providers.	The bill has the potential to reduce the number of deaths from opioid overdose.
(H.B. 1449, 2015) Natural resources and energy	Intended to prevent and improve the state’s ability to respond to oil spills.	The bill could decrease water and public health risk factors related to oil spills, particularly for communities of color, low-income communities, and populations with lower levels of educational attainment.
(S.B. 5346, 2015) Health care and social services	Would require personal emergency response system companies to provide the location and known medical conditions of their costumers when requested by first responders during an emergency.	The bill could improve health outcomes during an emergency for older adults, individuals with disabilities and chronic conditions, and individuals with limited mobility.
(S.B. 5870, 2015) Health care and social services	Would expand the list of acts that constitutes unprofessional conduct by a licensed health care provider to include performing sexual orientation change efforts on a patient under the age of 18.	The bill could mitigate harms and improve health outcomes among LGBTQ patients.
(H.B. 1674, 2015) Criminal justice	Would provide the Department of Social and Health Services with custody of youth convicted as adults who expected to complete their term of confinement before their 21st birthday (and Department of Custody if they finish after their 21st birthday); Would require youth convicted as adults to access the same services and programming as youth convicted in juvenile court.	The bill has the potential to reduce recidivism for youth offenders, which could lead to a decrease in health disparities for this population.
(S.B. 6029, 2015) Labor and employment	Would increase the state minimum wage annually with the rate of inflation, and increase at inflation plus 3% during years when per capita personal income increased over the year before and when it was higher than the per capital personal income for the country.	The bill could increase income and improve related health effects for low-wage workers.
(H.B. 1356, 2015) Labor and employment	Would require employers with five or more full-time employees to provide paid sick and safe leave to employees for reasons related to closure of the employee's place of business or childcare, and purposes related to domestic violence, sexual assault, and stalking.	The bill has the potential to improve financial security, decrease the transmission of communicable disease, improve health outcomes, and decrease health disparities by income, educational attainment, race and ethnicity, and geography.
(H-0915.3/15 draft, 2015) Labor and employment	Would require the Department of Labor and Industries to develop rules creating workload standards for employees performing commercial janitorial services.	Reducing workload and rushing among commercial janitors would likely decrease workplace injury disparities by race/ethnicity, English proficiency, country of origin, education, and income. It is not clear from available studies if the specific standards required in the bill would lead to decreased work intensity and rush.
(S.B. 5459, 2015) Labor and employment	Would require employers to annually provide twelve weeks of family and medical leave insurance to eligible employees for the birth or placement of a child and for a family member’s serious health condition, plus 12 weeks for the employee’s own serious health condition.	The bill could improve financial security; to improve maternal, child, and family health; and to decrease health disparities by income, educational attainment, and race/ethnicity.
(Budget Proposal, 2015) Labor and employment	The 2015–2017 Individual Provider Home Care Contract is a tentative agreement between the State of Washington and SEIU 775 regarding individual providers who have contracted with the Department of Social and Health Services (DSHS) to provide personal care, respite care, or residential services.	Funding would likely improve health outcomes for home care providers, thereby decreasing health disparities by race/ethnicity and income in the state.
(S.H.B. 1458, 2015) Agriculture, food, and drug	Would change the minimum age at which a person may purchase and possess cigarettes, tobacco products, and vapor products from 18 to 21 years old; Modifies the definition of a “vapor product”.	The bill would likely decrease use of tobacco and vapor products among youth and young adults, thereby improving health outcomes. It is unclear how the bill would impact health disparities, though some evidence suggests that the effect on disparities may be neutral.
(H.B. 2307, 2016) Labor and employment	Would require employers to provide reasonable accommodation in employment for pregnancy, childbirth, or pregnancy-related health conditions, unless the accommodation would impose an undue hardship on the employer.	The bill has potential to improve maternal and child health and to decrease health disparities by race/ethnicity and income.
(H.B. 1865, 2016) Education	Would require every board of school directors to provide for screening for near vision acuity in addition to screening already required for distance vision acuity.	The bill has potential to increase the number of students who have near-vision problems detected and treated, which in turn has potential to improve educational, income, and health outcomes for these students, but unclear how the bill would impact treatment and long-term outcomes.
(H.B. 2313, 2016) Agriculture, food, and drug	Would prohibit selling or giving tobacco or vapor products to a person under the age of 21; Modifies the definition of a “vapor product”.	The bill would likely decrease use of tobacco and vapor products among youth and young adults, thereby improving health outcomes. It is unclear how the bill would impact health disparities, though some evidence suggests that the effect on disparities may be neutral.
(S.B. 6149, 2016) Labor and employment	Would require employers to provide reasonable accommodation in employment for pregnancy, childbirth, or pregnancy-related health conditions, unless the accommodation would impose an undue hardship on the employer.	The bill has potential to improve maternal and child health and to decrease health disparities by race/ethnicity and income.
(H.B. 2969, 2016) Agriculture, food, and drug	Would impose a 45% tax on the taxable sales prices of vapor products; create a distributor and retailer license to distribute or sell vapor products in Washington; and require that, at a minimum, 3% of the revenues collected from the vapor products tax be appropriated from the general fund to the Cancer Research Endowment Fund Match Transfer Account.	The bill would likely decrease vaping rates in Washington State, thereby improving health outcomes and decreasing health disparities by socioeconomic status in the state.
(H.B. 2986, 2016) Health care and social services	Would create a premium assistance program for low-income Pacific Islanders living in Washington state under a compact of free association (COFA) to purchase health insurance through the health benefit exchange. The bill would also establish an advisory committee that would lead the development, implementation and operation of the program.	The bill has the potential to increase access and utilization of health care services, which may result in improved health outcomes and reduced health disparities, by increasing the number of COFA residents enrolled in a qualified health plans.

Source**:** Authors' analysis of HIR findings, bill summary, legislation year, and sector that the HIR applied to, based on data from http://sboh.wa.gov/OurWork/HealthImpactReviews for the defined study period

Twenty legislators, including 14 representatives and 6 senators, requested the HIRs. Of these 20 legislators, three were Republicans and 17 were Democrats. The primary sponsors of the proposals submitted for HIRs were Democrats (87%). Of the 36 HIRs in the sample, four (11%) were requested by Republicans compared with 32 (89%) requested by Democrats. Of the 20 legislators who requested HIRs, 40% requested more than one HIR, and the maximum number of HIRs requested by a legislator was nine.

**Fig. 2:**
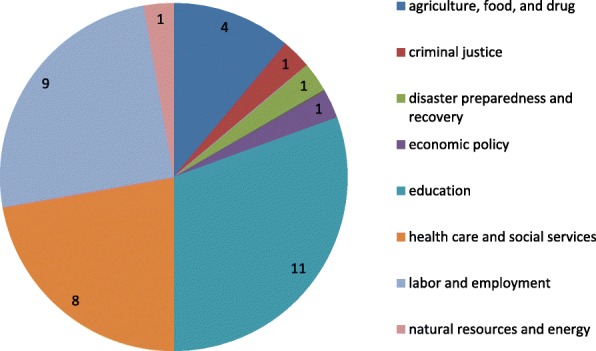
Number of HIRs in the sample, by Sector (*N* = 36). Source: Analysis of data from a database based on information from http://sboh.wa.gov/OurWork/HealthImpactReviews for the defined study period

As shown in Fig. 2, HIRs covered a range of sectors including: education (*n* = 11); labor and employment (*n* = 9); health care and social services (*n* = 8); agriculture, food, and drug (*n* = 4); criminal justice (*n* = 1); economic policy (*n* = 1); disaster preparedness and recovery (*n* = 1); and natural resources and energy (*n* = 1).

The HIRs in the sample examined the effects of the proposed legislation on a wide array of health determinants such as income, youth recidivism (as connected to involvement in the criminal justice system), access to culturally appropriate care, educational attainment, and water quality, and on health outcomes including mental health, vision problems, oral health, and communicable disease.

### Qualitative data

#### Conducting the HIRs: role of the Board of Health (BOH)

The BOH conducts all HIRs. During the study period, the staffing primarily involved one full-time person and occasionally a second staff person, depending on funding. In interviews, all of the individuals involved in conducting HIRs expressed that while having staff trained in public health is desirable, the key skills required to work on HIRs include experience conducting literature reviews and strong written and oral communication. The BOH also felt that having staff that understands equity is important because HIRs are used to determine the likely impacts of a proposal on health disparities.

Nearly all requests to complete an HIR were received during the legislative session; although a few have been received during the interim, which allows an HIR to be completed without additional time constraints. To support the implementation of HIRs, the BOH staff engaged in outreach to legislators and staff to increase awareness, testified on the HIR findings during bill hearings, and attended community meetings to share HIR findings.

Some of the respondents perceived the BOH as an unbiased producer of HIRs. Because of their objectivity regarding the science, the BOH was described as the right team to generate HIRs. One interviewee described HIRs “as reliable and credible as the people who are doing them.” However, a few of the respondents did not share this perspective. One of the respondents felt that the methodology was unclear, which made this stakeholder question the credibility of the data being produced by the BOH, although the respondent was careful to note that the issue was not with the BOH:

“… The problem is that they take current reports, current health reports or impact and studies that are out there, and that’s what they base their Health Impact Review on. So, if you have a field that is heavily studied, there will be a lot of reports to look at. If you have a field that is not heavily studied, there won’t be a lot to look at. And then, but they don’t go outside of those reports for their final, well, how it is an impact …. I’m not putting blame on them. I just, I think [it’s] the process.”

#### Perceptions of the structure and content of HIRs

Most interviewees found the format of the HIRs useful, especially the pathway diagrams linking the bill topic to health. Some interviewees felt that the list of references provided by BOH is helpful and adds to the credibility of the HIR. Interviewees also felt that having strength of evidence ratings to describe the quality of the research in the HIR was useful; however, some felt that this information needed to be more prominent rather than “buried in the report.”

HIRs do not provide recommendations, and each of the stakeholders who were interviewed expressed that this was important for objectivity and credibility as well as that it would be inappropriate for the BOH to provide them. This perspective was well captured by a legislator who shared that, “HIR lays out evidence and the elected official has been elected to make the decision about what to do based on the evidence.”

Some interviewees perceived that HIRs are limited since they only present evidence based on a review of available literature and are not known for “data crunching.” Some legislators want quantifiable data in the HIR, but BOH staff noted that this is not always possible due to the time and resources needed to provide such information. When they can, the BOH staff does include a section in the HIRs on the magnitude of the potential health impacts [9], which was also described in the interviews.

#### Perceptions of HIRs’ role in policymaking

HIRs were viewed by advocates and some legislators as providing the best available public health data that can be shared during bill hearings, cited during meetings, included in white papers, and used by advocates. These supporters viewed them as a way to provide

“… [a look at] the policy from the social justice health equity lens.”

Several interviewees perceived HIRs as useful because they summarize the evidence linking a proposed policy and health, especially for bills outside of the health committee. One legislator’s perspective illustrates the value of having HIRs for these bills:

“… bills coming through the health committee, they expect us to already be thinking about the health impacts. Whereas the other committees, you wouldn’t automatically think about the connections with health.”

One respondent said that HIRs “removed the politics” from the debate and they “just provided data.” HIRs were described as helping provide needed evidence. One legislator expressed this perspective when she said it is “… good to have the evidence to think about how it [the bill] impacts health equity.” Several interviewees shared the sentiment that HIRs clearly lay out the evidence of the impacts of a bill on health and health equity, which is helpful to individuals passionate about those outcomes.

Other respondents offered a contrary perspective and perceived HIRs as an advocacy or political tool. Some of those interviewed perceived that legislators requested HIRs for bills they thought would be good for health in order to gather the evidence to support their position. One respondent said, “The legislator who wants the bill to pass [or] sponsors the bill loves to have it.” One lobbyist shared the following:

“So if you know that the studies are out there and you think it was going to benefit your program, you ask for the health impact [review]. If you don’t like what’s out there, you don’t ask for the health impact [review] … so it’s becoming more a political tool than any kind of real analysis of what’s going on.”

From interviews with legislators and staff involved with the development of HIRs, HIRs were originally intended to help legislators understand and explore the potential health impacts of a range of proposed bills. In practice, to date, they have most often been requested by legislators to help provide general evidence to support their own bills. In fact, one legislator said that she had not “thought about using HIRs for [a] con position,” and only sees them requested for “when they are trying to get something passed.”

#### Perceived impacts of HIRs

The BOH does not systematically evaluate HIRs; thus, no data were available to objectively describe the impact of HIRs completed during the study period on the policy process. However, the BOH reported that they collect feedback from legislators, staff, and other stakeholders on the use of HIRs. Based on this feedback, staff has been told about the value of the data included in HIRs.

When interviewees were asked about the impacts of HIRs, they mainly described how HIRs included data that were often cited during bill hearings. One advocate interviewed for this research emphasized this point when she shared that her organization cited data from an HIR in testimony supporting the bill. HIRs were also described as being part of references in other documents related to proposed legislation. For example, one respondent reported that in July 2016 a member of the Seattle City Council wrote a white paper on the topic of conversion therapy as part of efforts to sponsor an ordinance to prohibit providers from using this practice, and referenced an HIR the BOH conducted on the issue during the 2014 session. The ordinance was unanimously approved in August 2016.

One interviewee described how an HIR also helped illuminate “how much the budget cuts would affect different populations” and was perceived as an important tool during budget negotiations to prevent severe cuts for health-supportive programs. About this particular HIR, one legislator recalled the following:

“That is one [HIR] I remember got used quite a bit in the discussion, because we were trying to prioritize what should we cut, how it would affect different populations, and we were able to show how it would negatively impact people. [HIRs] could be useful on another budget.”

#### Barriers to expanding use of HIRs and potential solutions

Several barriers to expanding HIRs in Washington were described. Several interviewees felt that legislators were largely unaware of HIRs and the process to have one completed. Interviewees also felt that “people don’t ask for them [HIRs] because they don’t realize the breadth of the scope” or because they “believed that HIRs were only for bill sponsors.” The broad definition of health is not obvious, and some might think that HIRs focus on health care, which is another barrier. Regarding this point, one respondent said that until he learned more about HIRs, he “never gave it much thought because [he is] not interested in health care.” He was not aware that HIRs do not focus solely on health care.

Several interviewees felt that another barrier to their expanded use is that only a legislator or the governor may request one. HIRs were perceived as potentially having greater effects if they could be requested through an open process where members of the general public could request one. Several advocates and lobbyists interviewed for this research described how they were aware of HIRs and asked a legislator to request one for a bill.

Interviewees also perceived challenges with the HIR approach and methodology, which may also limit their expanded use. Some interviewees reported being skeptical of HIR findings because it is unclear how the BOH accounts for uncertainty in the findings from the literature. Because of this uncertainty, some stakeholders feel that HIRs should not be completed if there is insufficient evidence. Another challenge related to the methods is that many of the connections between the policy and health involves indirect associations. One lobbyist said that it is problematic to complete HIRs based on a “cursory connection between health and the topic … [you] cannot say [you are] going to improve the health of everyone by making a policy change.” Finally, regarding the methodological approach, some HIRs include national-level data, which interviewees felt are not as useful as those with state and local sources of data.

Finally, some interviewees perceived HIRs as a political tool, which raised questions about the objectivity of the analysis and was perceived as hindering more widespread use. Several interviewees felt that HIRs could be used more if they were provided for each bill and not only when they were requested. If there was an independent way of selecting bills for HIRs, the belief is that it would not be viewed as a political or advocacy tool, since several respondents believe that legislators mainly request them when they need data to support a bill.

## Discussion

This manuscript presents the first independent exploration of the Washington state HIR process, which is a novel strategy for integrating health considerations into the state’s policymaking process. HIRs are used to describe the potential health implications of bills, especially those in non-health sectors, such as bills related criminal justice, education, and social services, as well as budget proposals. HIRs reflect the diversity of HiAP approaches employed worldwide [12]. This research extends the existing literature regarding implementation of HiAP and has important implications for efforts to advance HiAP in the U.S. and abroad.

HIRs were perceived by some legislators and advocates as providing useful data for the policy debate. Several researchers have described voluminous, inaccessible data as a barrier to uptake of research by policymakers [13, 14]. Findings from additional studies also support a desire of policymakers for concise, clearly translated information that is relevant to the policy context [15, 16]. Because HIRs are created to include the best available evidence in a succinct way, highlighting a broad range of potential health impacts, they hold promise for responding to this barrier to uptake and desire for concise information. There is a balance between providing sufficient detail and the length of the HIR, and notably some respondents sought additional information on the methods used to formulate the HIR.

Despite the gradual increase in the number of HIRs requested annually during the study period, the interview data from this study suggests that limited awareness of the HIR process continues to be an important barrier to their use. Currently, HIRs are completed when there is a request, and, no request has been turned down because of insufficient capacity during sessions when the BOH had funding for the program. However, there were several occasions when a request was received or the BOH was approached by a legislator to discuss a request, and there were other HIRs in the queue. After providing a date for when it would be completed, some of these requestors indicated it would not be sufficiently timely, so they opted not to make a formal request. As awareness of HIRs continues to grow, there may be increased demand, which is important for the BOH to monitor to ensure they are adequately staffed and able to respond to requests in a timely manner, which may require additional resources from the legislature.

Funding and dedicated staff have been identified as facilitators of HiAP implementation in the literature base examining HiAP approaches in several countries and regions [6, 12, 17]. The descriptive data from the HIR review identified that when funding for the reviews and affiliated staff was suspended during the recession, the BOH was unable to conduct any HIRs. In addition, prior literature highlights the importance of a shared vision and dedication to HiAP implementation across sectors [6, 12]. It is possible the limited awareness of the HIR process identified through this study reflects a broader gap in cross-sector understanding of and commitment to HiAP strategies, or perhaps reflects the perspective shared by some people included in this research about the definition of health. Several respondents in this research shared that they essentially dismissed HIRs because they did not think the bill they were working on connected with health care.

In creating the shared vision described earlier in this section, Ollila has written that, for HiAP, getting health on the policy agenda is one important component for policy change as specified by Kingdon’s Multiple Streams Model [18]. Several interviewees noted that HIRs include important data that showed how health determinants impact health disparities and health outcomes. Drawing on Kingdon’s model and Ollila’s application for HiAP, HIRs are generating important data connecting decisions to public health data and, by conducting policy analyses from a health lens, are helping raise and sustain health issues on the policy agenda in Washington state [18]. Educating the public, policymakers, and stakeholders so they understand the various ways that social, economic, and environmental determinants impact health and equity, and resulting disparities, and how policies across a range of sectors can affect health and equity as part of creating a shared vision, could bolster HIR implementation efforts to support HiAP.

HIRs in this sample were mainly requested by Democrats (17 of the 20 requestors), which may raise questions about their broad appeal and utility. However, data from the 2017 session, not included in this study, indicate that roughly half of the requests were made by Republicans, suggesting that the HIR program is evolving over time. While this specific analysis did not explore the potential impacts of political party on requests or perceptions of HIRs, anecdotally Democrats seemed to have more favorable perspectives. Future research may benefit from exploring potential differences by political ideology to determine barriers and facilitators to their widespread use.

Although the BOH intends for HIRs to be objective, since the BOH is part of the Executive Branch, some do not perceive it as an independent agency. Based on the interviews, HIRs were perceived by some as a tool for supporting a bill and less often to inform a legislator before deciding his or her position. This perception by some respondents, that HIRs are a political tool, also presents a barrier to their expanded use. Findings from a cross-case analysis of successful HiAP implementation in Sweden, Quebec, and South Australia show that awareness-raising of HiAP efforts alone, without employing other strategies such as highlighting shared benefits, is not effective in building buy-in among non-health sector partners [19]. One way to address this barrier to the expanded use of HIRs is for BOH to increase education on HIRs (focusing on their purpose, methodology, and shared benefits across sectors and among policymakers) and to ensure that legislators and staff know that HIRs can be requested for any bill, even if they are not the lead sponsor, do not support the bill, or do not yet have a position on the bill.

HIRs are sometimes confused with HIAs, which are also used to advance HiAP. HIRs and HIAs are two tools jurisdictions can employ to inform policy-making and are not mutually exclusive. HIRs are different from HIAs in that they focus exclusively on proposed legislation as opposed to other decision types, are typically conducted within a 10-day timeframe, rely exclusively on the existing evidence base, and involve minimal stakeholder engagement [9, 20, 21]. Because they can be completed within a shorter timeframe than HIAs, HIRs can be used during rapid legislative sessions to provide policymakers with information about the potential impacts for health and health disparities. Jurisdictions that are exploring the use of HIRs, HIAs, and other HiAP approaches may want to consider their goals in implementing such approaches, the policymaking timeframe, and the appropriate level of stakeholder engagement to determine which tool is most appropriate for a given decision-making context.

Although this research generated new and important findings on HIRs in the context of advancing HiAP, there are some limitations of this research. While a diverse sample of individuals participated in interviews, some respondents declined to participate or never responded to multiple requests; thus, response bias is a concern. Also, while snowball sampling is an effective way to gather additional interviewees, respondents may suggest individuals who have similar views to their own, which may have biased the nonrandom sample used for this research. To address this, the team asked participants to suggest people who both supported and were critical of HIRs. Thus, while these strategies may have resulted in a biased sample, the sample did indeed include people who offered both perspectives.

## Conclusions

HIRs are a potentially effective strategy for including public health data and science in the policy process to mitigate potential harms to health and reduce health disparities and inequities. BOH, largely viewed as a credible source of HIRs, could address challenges to expanding their use by increasing awareness among legislators and staff about their objectivity, how they conceptualize health, and how they can be requested for any bill and highlighting shared benefits of HIRs that appeal to the interests of policymakers focused across diverse sectors. As the number of HIRs continues to grow, future research should continue to examine the impact that HIRs, especially those completed after this research ended, has on state policymaking.

## Additional file

Additional file 1: Key Informant Interview Guide Description of data: interview guide 2. (PDF 103 kb)

## Data Availability

The datasets used and/or analyses during the current study are available from the corresponding author on reasonable request.

## Abbreviations

BOH: Board of Health
HIRs: Health Impact Reviews
