# Supplementary material for: Considering health and health disparities during state policy formulation: examining Washington state Health Impact Reviews
Source: BMC Public Health. 2019 Jul 3;19:862. doi: 10.1186/s12889-019-7165-7 (PMC6610847; doi:10.1186/s12889-019-7165-7)
Supplement: Supplementary file 1 — Key Informant Interview Guide Description of data: interview guide 2. (PDF 103 kb) [file 12889_2019_7165_MOESM1_ESM.pdf]

JOHNS HOPKINS BLOOMBERG SCHOOL OF PUBLIC HEALTH

KEY INFORMANT INTERVIEW GUIDE

(LEGISLATOR OR STAFF/EXECUTIVE BRANCH/BOARD OF HEALTH/STAKEHOLDERS)
*The questions included in this protocol will serve as a general guide for the interviewer. Specific follow-up questions may vary

PI Name: Keshia Pollack, PhD, MPH
Study Title: Exploring the Role of Health Impact Reviews in Washington State Policymaking
IRB No.: 7129
PI Version No./Date: Version 1/April 12, 2016

Prior to starting the interview
Explanation of our research
Reason for the interview
Review oral consent

CONFIRM PERSON'S ROLE/POSITION

Experience with HIRs

Can you tell us the various ways that you have interfaced with HIRs (requested one on a bill, read one, cited the findings, etc.)? [Probe based on response]

Perceptions of HIRs

- What are you overall impressions of HIRs? [Probe]
- Do you think that HIRs have impacted policymakers understanding of how legislative and budgetary proposal could affect public health or health disparities? If yes, why/how and if not, why not?
- Do you think that HIRs have impacted the policy process? If yes, why/how and if not, why not?
- To what extent has the HIR process resulted in challenges or any unintended consequences?

- How might HIRs be even more impactful? What changes could be made to the process to address any challenges or increase their utility?

Conclusion

- Is there anything you would like to tell me about HIRs that we haven't already discussed?
- Who else should we be talking with in order to learn about HIRs?
